# Genome characterization of BI2 subcluster *Streptomyces scabiei* bacteriophages GoblinVoyage and Doxi13

**DOI:** 10.1128/mra.00581-24

**Published:** 2024-08-20

**Authors:** Hanna Jin, Nihal K. Chana, Annie L. Tang, Paramjit Kaur, Brishti Lamichhane, Sze Ching Leung, Diane Scheiderer, Vighnesh V. Sivaprakasam, Dannah T. Marcelino, Gregory J. Hull, Toma M. Kamara, 2023 UMBC Phage Hunters, STEM BUILD at UMBC Cohort 7, Maria C. Guimaro, Steven M. Caruso

**Affiliations:** 1 Department of Biological Sciences, University of Maryland Baltimore County, Baltimore, Maryland, USA; 2 College of Natural and Mathematical Sciences, University of Maryland Baltimore County, Baltimore, Maryland, USA; Department of Biology, Queens College, Queens, New York, USA

**Keywords:** bacteriophage, *Streptomyces*, plant pathogen, phytopathogens, soil microbiology, bacteriophage genetics, actinomycetes, genomes

## Abstract

We present the bacteriophages GoblinVoyage and Doxi13, siphoviruses isolated on *Streptomyces scabiei* RL-34. They belong to the BI2 cluster and have genomes consisting of 60.9% GC content with identical 3’ end sticky overhangs. The genome lengths of GoblinVoyage and Doxi13 are 43,540 bp and 43,696 bp, respectively.

## ANNOUNCEMENT

*Streptomyces scabiei* is a Gram-positive phytopathogen, primarily responsible for the common potato scab disease (1). Genomic characterization of bacteriophages that infect *S. scabiei* reveals roles that they may serve to address the deleterious effects of the infection.

**Fig 1:**
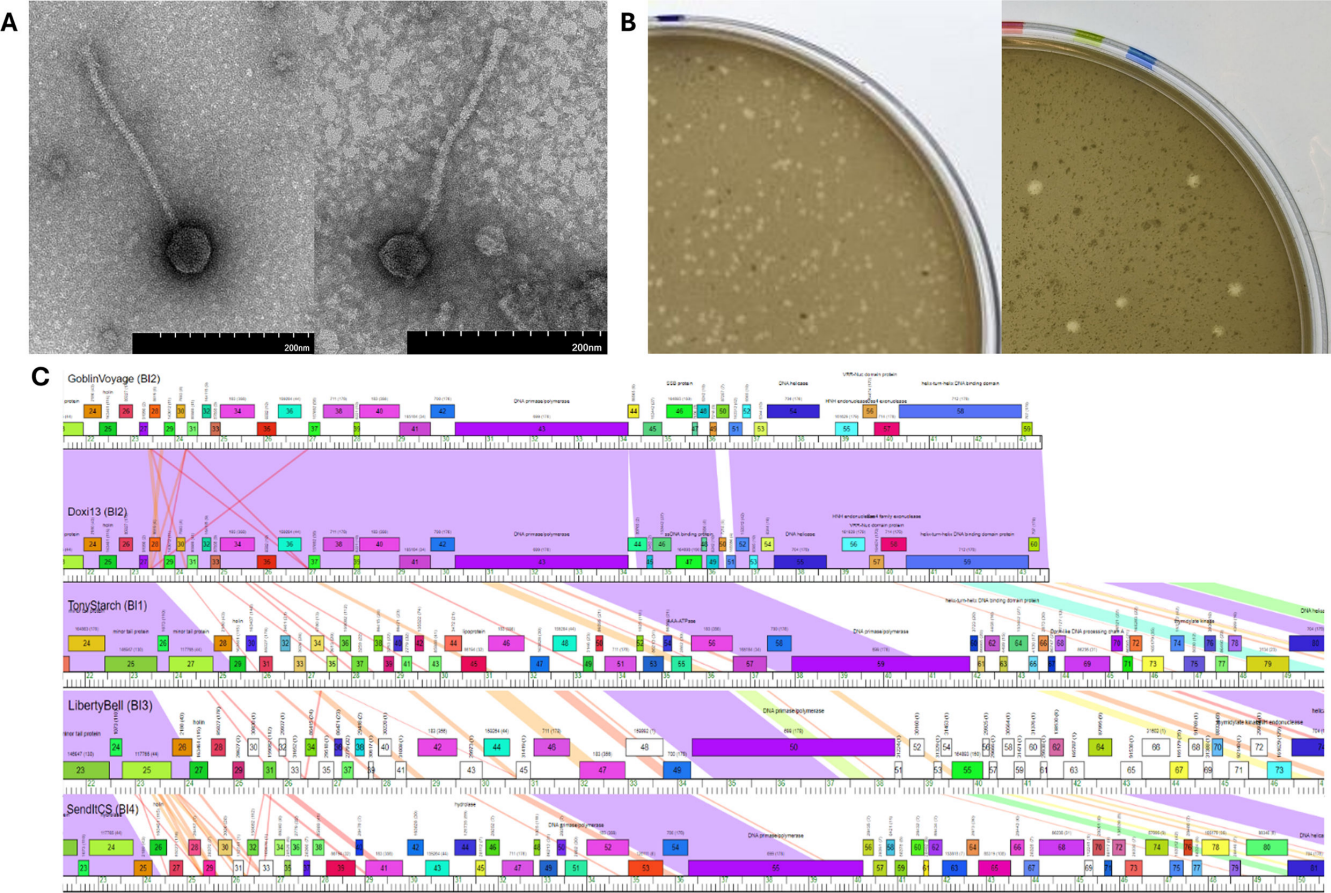
Morphological and functional characterization of BI2 phages GoblinVoyage and Doxi13. (**A**) Representative transmission electron microscopy images of GoblinVoyage and Doxi13. The fresh lysate sample was stained with 2% uranyl acetate. TEM imaging revealed GoblinVoyage and Doxi13 are siphoviruses with flexible and noncontractile tails. GoblinVoyage (left) has a capsid diameter of 58 nm (*n* = 3, SD = 2.5 nm) and tail length 261 nm (*n* = 3, SD = 1.53). Doxi13 (right) has a capsid diameter of 52 nm (*n* = 3, SD = 1.5) and tail length 248 nm (*n* = 3, SD = 12.56). All measurements were taken using ImageJ v1.54i (3). (**B**) Plaque morphology of GoblinVoyage and Doxi13 (left to right). Both phages showed turbid plaques with an average diameter of 1.10 mm (*n* = 30, SD = 0.337) and 1.60 mm (*n* = 16, SD = 0.46), respectively. Doxi13 plaques were observed in *S. mirabilis*. Plaques were measured after approximately 24 to 48 hours of incubation at 30°C. All measurements were taken using ImageJ v1.54i (3). (**C**) Comparison of GoblinVoyage and Doxi13 genome size and GoblinVoyage to other BI subcluster phages. From top to bottom: GoblinVoyage (BI2), Doxi13 (BI2), TonyStarch (BI1), LibertyBell (BI3), and SendItCS (BI4). All phages are compared to GoblinVoyage from approximately 21,500 bp. All phages selected are representative or the only member of their subcluster. Phages from subclusters BI5, BI6, and BI7 show similar results and are not included. Image produced using Phamerator vActino_Draft 558 (4).

Samples of bacteriophages GoblinVoyage and Doxi13 were collected from soils using *S. scabiei* RL-34 as a host (Table 1). All protocols were derived from the Science Education Alliance-Phage Hunters Advancing Genomics and Evolutionary Science (SEA-PHAGES) *Phage Discovery Guide* (2). Soil samples were combined with phage buffer (10 mM [Tris pH 7.5], 10 mM MgSO_4_, 68 mM NaCl, and 1 mM CaCl_2_) and passed through a 0.22-µm filter. Then, 500 µL of the filtrate was added to 250 µL of a 48-hour *S*. *scabiei* culture, incubated for 10 minutes, combined with 3 mL of tryptic soy soft agar (BD), then plated onto nutrient agar plates (BD Difco) with *Streptomyces* phage supplement (10 mM MgCl_2_, 8 mM Ca(NO_3_)_2_, 0.5% glucose) and incubated for 24–48 hours at 30°C. Phages underwent a minimum of three rounds of plaque purification. In short, picked plaques were serially diluted tenfold and used for plaque assays on the host. The fresh lysate was harvested from plates containing near-confluent lysis for imaging and DNA isolation. Negative-stained transmission electron microscopy revealed siphoviral morphotypes for both GoblinVoyage and Doxi13 (Fig. 1A). Both phages demonstrated a similar plaque morphology (Fig. 1B). Neither phage produced discernable lysogens when tested by spotting dilutions of the lysate on *S. mirabilis* and incubating for 7 days at 30°C, suggesting they followed a lytic infection cycle.

**TABLE 1:** Properties of *Streptomyces* bacteriophages GoblinVoyage and Doxi13

Phage characteristics	GoblinVoyage	Doxi13
Isolation host	*Streptomyces scabiei* RL-34	*Streptomyces scabiei* RL-34
Sample type	Dry, dusty soil	Dry, sandy soil
Sample location [GPS]	Golansville, Virginia, USA [37.980611 N, 77.491896 W]	Laurel, Maryland, USA [39.15222 N, 76.96722 W]
Approximate shotgun coverage	1,166 bp	10,298 bp
Total number of reads	357,901	3,008,314
Approximate fold coverage	1,233 x	10,327 x
Genome length	43,540 bp	43,696 bp
GC% content of phage	60.9%	60.9%
Genome end type	3' sticky overhangs	3' sticky overhangs
Character of genome ends	5′-CGCCGCCCT-3′	5′-CGCCGCCCT-3′
Identified protein coding genes (assigned a function)	59 (29)	60 (21)
Identified tRNA encoding genes	0	0

Phage DNA was extracted from the freshly prepared crude lysate using the Promega Wizard DNA cleanup kit and sequenced at the Pittsburgh Bacteriophage Institute using the NEB Ultra II Library Kit and an Illumina MiSeq with v3 reagents with 150-bp single-end raw reads. The sequencing results are described in Table 1. Newbler v2.9 (5) and Consed v29 (6) were used for assembly, quality verification, and end determination as described (7).

Genome annotation was completed using DNA Master v5.23.6 (8) with internal Glimmer v3.02 (9) and GeneMarkS v2.5 (10, 11) programs. GeneMark.hmm v2.5p (10, 11) was used to confirm these annotations with *S. scabiei* 87–22 as the species. Putative functions were assigned by assessing amino acid sequence homology using NCBI BLASTp v2.15.0 (12) and Conserved Domain Database v3.19 (13), structural homology with HHpred v57c87 (minimum probability: 90%) (14, 15), and synteny via Phamerator vActino_Draft 558 (4). Default parameters were used for all software, unless otherwise specified. The phages were assigned to cluster BI2 in the Actinobacteriophage Database (PhagesDB) based on gene content as described (16, 17) and the genus *Scapunavirus* (18). Genomic characterization and annotation results are reported in Table 1.

Annotations of GoblinVoyage and Doxi13 failed to identify genes consistent with a temperate phage, agreeing with the earlier virulent designation and classification of the cluster in PhagesDB. Genomic comparison of phages from BI subclusters shows that BI2 phages have smaller genomes, differing by approximately 10 kbp, including multiple small genes in the center of the genome (Fig. 1C).

## Data Availability

GoblinVoyage is available at GenBank with accession number PP725412 and at SRA with accession number SRX24123906. Doxi13 is available at GenBank with accession number ON970617 and at Sequence Read Archive (SRA) with accession number SRX20165790.
